# E2F1/2/7/8 as independent indicators of survival in patients with cervical squamous cell carcinoma

**DOI:** 10.1186/s12935-020-01594-0

**Published:** 2020-10-12

**Authors:** Chang Yang, Zhao-Cong Zhang, Tian -Bo Liu, Ye Xu, Bai-Rong Xia, Ge Lou

**Affiliations:** Department of Gynecology Oncology, Harbin Medical University Cancer Hospital, Harbin, 150086 P.R. China

**Keywords:** E2F transcription factors, Cervical cancer, Prognostic value, Bioinformatic analysis

## Abstract

**Background:**

Cervical cancer is the second leading cause of death in women 20–39 years old. Because coverage for cervical cancer screening is low, and the vaccination rate of human papillomavirus (HPV) is poor in some countries, potential markers to detect the disease at early stages are needed. E2F transcription factors (E2Fs) are a family of transcription factors that function in cell proliferation, differentiation, apoptosis, and tumorigenesis. As abnormal activation and regulation of E2Fs are related to tumor development and poor prognosis, we performed bioinformatic analyses and in vitro assays to evaluate the role of E2Fs in cervical cancer.

**Methods:**

Transcriptional expression of E2Fs was initially evaluated in silico using ONCOMINE and Gene Expression Profiling Interactive Analysis (GEPIA), followed by evaluation of E2F1/2/7/8 protein levels using immunohistochemistry in 88 patient tissues. E2F2 and E2F7 mRNA levels were measured by RT-qPCR. LinkedOmics and Metascape were used to predict functions of E2Fs, and in vitro experiments were performed to assess the tumorigenic role of E2F2 and E2F7.

**Results:**

In silico analysis showed that E2F1/2/7/8 were significantly overexpressed in cervical cancer, findings which were confirmed at the protein level using immunohistochemistry. Further, upregulation of E2F1/2/7/8 was associated with different clinicopathological prognostic factors, including positivity for lymph vessel invasion and deep invasion of cervical stroma. Increased expression of E2F1/2/7/8 was also related to shorter overall survival (OS) and disease-free survival (DFS) in patients with cervical cancer. Using multivariate analysis, we confirmed E2F1/2/7/8 as independent prognostic factors for shorter OS of patients with cervical cancer. Finally, in vitro experiments showed that E2F2 and E2F7 are involved in cell proliferation and migration and cell cycle regulation in both HPV-positive and HPV-negative cervical cancer cells.

**Conclusions:**

E2F1/2/7/8 may be prognostic biomarkers for survival of patients with cervical cancer. E2F2 and E2F7 are involved in cell proliferation, migration, and cell cycle in both HPV-positive and HPV-negative cervical cancer cells.

## Background

According to the American Cancer Society, it is estimated that 13,800 cases of invasive cervical cancer will be diagnosed and 4,290 deaths from cervical cancer will occur in the United States in 2020 [1]. It is the second leading cause of death in 20- to 39-year-old women, responsible for 10 premature deaths per week in this age group globally [2]. The leading cause of cervical cancer is infection with human papillomavirus (HPV); thus, the incidence and mortality of cervical cancer varies widely with geographic location because of differences in HPV screening and vaccination programs [3]. The World Health Organization set a goal to eliminate cervical cancer in 2018 [2]; however, in China, cervical cancer screening was adopted in 2009 [3] and it was only in 2016 that the HPV vaccine was approved [4]. In addition, the population coverage rate for cervical cancer screening is only 21.4%, and the HPV vaccination rate is very poor because the vaccine has not been introduced into the national immunization program [5]. Because of the low cervical cancer screening rate, many patients at diagnosis are already at an advanced stage or have locally advanced cervical cancer. Despite provision of standardized initial treatment, including surgery, radiotherapy, and chemotherapy [6], there remains a high risk of recurrence and death in these patients. Therefore, the identification of new therapeutic target is critically important for the treatment of cervical cancer.

Members of the E2F family of transcription factors are expressed in a variety of tumors, having roles in proliferation, differentiation, apoptosis, and tumorigenesis; further, their abnormal activation and regulation are related to tumor development and poor prognosis [7–9]. There are eight members of the E2F family, termed E2F1–8, acting as either transcriptional activators (E2F1–3) or repressors (E2F4–8) [10]. They are expressed at low levels in normal tissue, but overexpressed in tumor tissue, making them attractive potential therapeutic targets [9–11]. Further, aberrant expression of E2F family members and their association with clinicopathological features and prognosis in patients with cancer have been demonstrated. In this study, we used bioinformatics to broadly investigate and obtain a deeper understanding of the relationship between E2Fs and cervical cancer. We determined gene expression at the mRNA and protein level and evaluated the clinical significance and independent prognostic value of E2F family members in our cohort. We also explored the biological functions and pathways of genes with expression patterns similar to those of E2Fs.

## Methods

### ONCOMINE analysis

ONCOMINE is a publicly accessible, online cancer microarray database aimed at facilitating research through the provision of genome-wide-expression data [12]. In the present study, we used ONCOMINE to determine mRNA levels of the eight different E2F family members and compare them between different tumors and matched normal tissues, Shan parameters: *p* < 0.01, fold change > 1.5, gene rank: 10%, and data type: mRNA.

### GEPIA dataset

Gene Expression Profiling Interactive Analysis (GEPIA) is an interactive web server designed to facilitate evaluation of mRNA expression data from The Cancer Genome Atlas and Genotype Tissue Expression projects [13]. GEPIA includes many features, such as profiling the relative transcriptional expression of genes of interest between tumor and normal tissues, analysis according to pathological stage, survival analysis, and similar gene identification. We used GEPIA to compare mRNA expression of the eight E2F family members in normal cervical tissues and cervical cancer tissues from patients in our cohort. Differences in transcriptional expression were determined by Student’s t-test and *p*-values < 0.05 were considered statistically significant. In addition, we identified a series of genes with expression patterns similar to those of E2F family members in cervical cancer using GEPIA.

### Patients and clinical tissue samples

In this study, 98 specimens archived on formalin-fixed, paraffin-embedded tissue blocks were obtained from January 2010 to December 2015 from the Harbin Medical University Cancer Hospital in Harbin, China. Study samples were from 88 cervical squamous cell carcinoma and 10 normal cervical tissues. All 88 patients in our study were diagnosed with cervical cancer and underwent a radical hysterectomy and pelvic lymphadenectomy. Subsequent treatment after surgery was conducted according to NCCN guidelines [14]. Normal cervical tissues were obtained from 10 women undergoing hysterectomy for benign uterine disease at the Harbin Medical University Cancer Hospital. No patient received immunotherapy, chemotherapy, or radiotherapy before surgery. Clinicopathological features of study participants are summarized in Additional file 1: Table S1. The median age of patients was 43 years (range: 22–74 years). Tissue classification was conducted based on World Health Organization tumor classification criteria. Staging was performed according to the 2009 modified International Federation of Gynecology and Obstetrics (FIGO) system [15].

### Immunohistochemistry

Immunohistochemistry (IHC) was performed using anti-E2F1, -E2F2, -E2F7, and -E2F8 antibodies following standard methodology. Tissue blocks were cut into 4-μm-thick sections and stained with hematoxylin. Tissue sections were deparaffinized using xylene and rehydrated in alcohol. Next, tissue sections were incubated at room temperature and 3% H_2_O_2_ for 10 min to remove endogenous peroxidase activity. A pressure cooker was used for antigen retrieval in citrate buffer (pH 6.0, 10 mmol/mL). After washing with phosphate-buffered saline (PBS), anti-E2F1, -E2F2, -E2F8 (1:100; Santa Cruz Biotechnology, Shanghai, China), and -E2F7 antibodies (1:100; Proteintech, Wuhan, China) were added, and samples were incubated overnight at 4 °C. Specimens were washed with PBS, and then incubated with horseradish peroxidase secondary antibody and DAB at room temperature for 45 min. Color was developed using 3,3′-diaminobenzidine tetrahydrochloride (Dako, Hamburg, Germany) for 10 min, and sections were then counterstained with hematoxylin for 2 min. Final dehydration in 100% ethanol was performed for 8–10 min and slides were dried to remove moisture.

Sections were scored blindly by two independent pathologists. The degree of immunostaining was based on staining intensity and percentage of cells stained. We quantitatively scored tissue sections using the following criteria: (a) percentage of immunoreactive cells: 0 (0%), 1 (0%–10%), 2 (11%–50%), 3 (51%–70%), and 4 (> 71%); and (b) staining intensity: 0 (negative staining), 1 (weak staining), 2 (moderate staining), and 3 (intense staining). Staining results were evaluated using both the percentage of positive staining and intensity of positively stained tumor cells. The sum of the intensity and extent scores was used as a final staining score (range, 0–7) [16]. E2F1/2/7/8 immunoreactivity scores < 4 was defined as low expression, whereas scores ≥ 4 was high expression.

### cBioPortal

cBioPortal is an online open-access website resource developed for comprehensive analysis of complex cancer genomics and clinical data [17]. In the present study, we explored the frequency of mutations in the eight genes of the E2F family, putative copy number variation from the Genomic Identification of Significant Targets in Cancer, and mRNA expression z-scores (RNASeq V2 RSEM) with a z-score threshold of ± 1.8. We also graphically displayed the relationships between gene mutations in E2Fs and overall survival (OS) and disease-free survival (DFS) of patients with cervical cancer using Kaplan–Meier plots. The difference of survival curves was analyzed with log-rank test and a *p*-value < 0.05 was considered statistically significant.

### LinkedOmics

LinkedOmics is a web resource for in-depth analysis of multi-omics data within and across 32 types of cancer [18]. We used Pearson’s correlation test in the “LinkInterpreter” modules to analyze Kyoto Encyclopedia of Genes and Genomes (KEGG) pathways of individual E2F genes, conducted by Gene Set Enrichment Analysis with a minimum number of genes (size) of 3,500 simulations, and a false discovery rate of 0.05.

### Metascape

Metascape is a newly developed online tool mainly for analyses of abundant annotations for thousands of genes, pathways, or process enrichment, and for protein–protein interactome network analysis [19]. We applied Metascape to gene ontology (GO) categories and KEGG pathway enrichment analysis of E2F family members and genes with expression patterns similar to those of E2F family members. Only terms with a *p*-value < 0.05, minimum count of 3, and enrichment factor > 3 were considered significant. Protein–protein interaction enrichment was performed using the molecular complex detection (MCODE) algorithm to identify neighborhoods where proteins are densely connected.

### Cell culture and transfection

The human cervical cancer cell lines HeLa (HPV-positive) and C-33 A (HPV-negative) were obtained from the Cell Bank of Chinese Academy of Sciences (Shanghai, China). Cells were cultured in Minimum Essential Medium (MEM; Corning, Shanghai, China) with 10% fetal bovine serum (FBS; Gibco, Shanghai, China) and penicillin–streptomycin at 37 °C with 5% CO_2_. A short hairpin RNA (shRNA) construct was designed targeting a specific sequence within human *E2F2* (5′-GATCCCGACTCGGTATGACACTTCGTTCAAGAGACGAAGTGTCATACCGAGTCTTTTTGGAAA‐3′) and *E2F7* (5′‐CCGGGTGCTGCCAGCCCAGATATAACTCGAGTTATATCTGGGCTGGCAGCACTTTTTG‐3′). A pPLK interference vector was used for construction of the sh-E2F2 and sh-E2F7 expression vectors.

293 T cells (Felbio, Shanghai, China) in logarithmic growth were seeded in 60-mm dishes until 90% confluence. The MEM medium was then changed without penicillin–streptomycin prior to transfection. GM easy Lentiviral Mix (Genomeditech, Shanghai, China) and recombinant lentiviral expression plasmid were added to Opti-MEM (Corning) in proportion, followed by the addition of HG Transgene Reagent, which was then mixed and added to 293 T cell culture medium. Medium was replaced with fresh medium 4–6 h after transfection. Supernatant of transfected 293 T cells was collected 48 h post-transfection and filtered with a 0.45 µm filter, aliquoted, and stored at −80 °C.

HeLa and C33A cells were cultured in 6-well plates to 50–70% confluency. Virus aliquot and polybrene were thawed slowly on ice. Polybrene was first added to the plate, followed by virus and overnight incubation. Next, cells were passaged and cultured. Puromycin was added to screen for stably infected cells. Knockdown efficacy was confirmed by quantitative reverse transcription polymerase chain reaction (RT-qPCR) and western blot analysis.

### Immunofluorescence assay

Localization of E2F2 and E2F7 was determined by immunofluorescence. HeLa and C-33 A cells were seeded in 24-well plates coated with polylysine and cultured at 37 °C. After cells became adherent, the original medium was removed and cells were washed with PBS three times. Cells were then fixed with 4% paraformaldehyde at room temperature in the dark for 15 min and washed with PBS three times, followed by a 15-min fixation in ice methanol at −20 °C. Sections were rinsed in PBS three times and blocked in 15% donkey serum for 45 min at 37 °C. Following removal of blocking solution, cells were subsequently incubated with primary anti-E2F2 (1:200, Santa Cruz Biotechnology) and primary anti-E2F7 (1:50, Proteintech) antibody at 4 °C overnight. The next day, rewarming was performed at 37 °C for 45 min and cells were washed three times with PBS. The respective anti-E2F2 or anti-E2F7 secondary antibody (200 μL of 1:400 dilution) was then added and incubated at room temperature for 2 h protected from light, followed by three washes with PBS. Coverslips were sealed with mounting medium containing DAPI and Hoechst 33258 and observed under a confocal microscope (Leica).

### RT-qPCR assay

Trizol reagent (Ambion, Shanghai, China) was added to cervical cancer tissues and cells from the human cervical cancer cell lines HeLa and C-33 A, and total RNA was extracted according to manufacturer’s instructions. After eluting with RNase-free water, RNA was stored at −80 °C until analysis. RNA quality was evaluated by spectrophotometer (Eppendorf), and then reverse-transcribed into cDNA using a reverse transcription kit (Vazyme, Nanjing, China). RT-qPCR was performed using a SYBR Green PCR kit. The RT-qPCR primers of *E2F2* and *E2F7* were designed from the Prime Bank Web site [20]. PCR cycling conditions were as follows: 95 °C for 2 min, followed by 94 °C for 20 s, 58 °C for 20 s, and 72 °C for 30 s for 40 cycles. GAPDH was used as the internal reference control. All RT-qPCR reactions were independently performed three times. Relative mRNA expression was determined using the comparative cycle threshold (2^−ΔΔCt^) method.

### Western blot assay

Total protein was extracted from cell lysates with a protease inhibitor. A BCA protein concentration measurement kit (Thermo Fisher Scientific, Shanghai, China) was used to determine protein concentration. Proteins were separated using 4–20% SDS-PAGE, and then transferred to a PVDF membrane, which was then blocked with 5% BSA for 1 h at room temperature followed by incubation overnight at 4 °C with the primary antibodies E2F2 (1:1000, Santa Cruz Biotechnology) and E2F7 (1:1000, Proteintech). Following six washes with TBST, the membrane was incubated with a second antibody (Abways, Shanghai, China) for 1 h, and relative protein expression was determined by electrochemiluminescence.

### Cell cycle assay

Cells in logarithmic growth (80–90% confluence) were collected, resuspension in complete media, and centrifugation (1000 *g* for 5 min). Following a rinse using pre‐cooled PBS at 4 °C and centrifugation at 1000 *g* for 5 min, ice-cold 75% ethanol was added and stored overnight at −20 °C. Following a wash with PBS, RNA was removed by the addition of 10μL RNase A Solution (Yeasen, Shanghai, China). Cells were stained with 10 μL propidium iodide (Yeasen) for 20 min in the dark. Detection was performed by flow cytometry (BD biosciences), using an excitation wavelength of 488 nm and emission wavelength of 585 ± 21 nm.

### Cell proliferation assay

Cells in the logarithmic growth phase were collected and resuspended after digestion with TrypLE (Gibco). Cells were seeded in 96‐well plates at a density of 2 × 10^3^ cells/well in 100 μL. CCK-8 reagent (10 μL, Yeasen) was added before cell activity was examined at 0, 24, 48, and 72 h. Cells were incubated in a 5% CO_2_ incubator at 37 °C for 2 h. Optical densities were measured using an microplate reader (Thermo Scientific, Shanghai, China). at wavelengths of 490 and 630 nm to determine cell proliferation.

### Wound healing assay

Logarithmically growing cells transfected with sh-E2F2, sh-E2F7, or negative control (sh-NC) were harvested, and seeded into a 6-well culture plate at a density of 5 × 10^5^ cells/well, which was incubated at 37 °C in 5% CO_2_ for 12 h. Once the cells grew to confluent monolayers, a 200 μL pipette tip was used to make a series of perpendicular scratches. Cells were then washed three times with PBS. Serum-free MEM medium was added, followed by incubation at 37 °C in 5% CO_2_. Images were taken at 0, 24, and 48 h with a microscope at 10 × magnification (Nikon).

### Transwell assay

HeLa and C-33 A cells were collected and re-suspended in serum-free medium (1 × 10^6^ cell/mL). A total of 100 μL of cell suspension (1 × 10^5^ cells/well) was seeded into the upper chamber of a 24-well Transwell plate (Corning Costar, Shanghai, China). In the lower chamber, 600 µL MEM medium supplemented with 20% FBS was added. Chambers were incubated at 37 °C in 5% CO_2_ for 24 h, and remaining cells were removed from the top of the permeable membrane using a cotton swab. Cells that had traversed the membrane were fixed in 95% ethanol for 15 min, stained with 0.1% crystal violet for 15 min, and excess dye was removed using PBS. Cells were observed using an inverted microscope (Nikon) and photographed. All experiments were performed in triplicate using three wells per experiment.

### Sphere-forming assay

Following TrypLE (Gibco) digestion and two washes in PBS, HeLa and C-33 A cells were resuspended in stem cell medium (Corning). Cells (1 × 10^3^) were seeded into ultra-low-attachment 96-well plates containing 200 µL of DMEM/F12 medium supplemented with 20 ng/mL EGF and 20 ng/mL basic fibroblast growth factor. Cells were cultured in an incubator at 37 °C in 5% CO_2_ for 5 days, after which size was measured (in µm) as the diameter of the microspheres and photographed using a microscope (Nikon).

### Statistical analysis

Clinicopathological variables were analyzed using Chi-square test with IBM SPSS Statistics for Windows version 22.0 (IBM Corp., Armonk, NY, USA). OS and DFS were calculated using Kaplan–Meier method. Univariate and multivariate Cox regression analyses were performed to estimate associations between mRNA expression of E2Fs, clinicopathological factors, and OS of patients with cervical cancer. A two-sided *p*-value < 0.05 was considered significant. Data of in vitro cellular function assays are expressed as the mean ± SD from three independent experiments. Statistical data analyses were performed by GraphPad Prism 7.0 (GraphPad Software, La Jolla, CA, USA). Differences between two groups were estimated using Student’s t-test, and two-way analysis of variance was used for comparisons of CCK-8 and cell cycle assay*.* A *p*-value < 0.05 was considered statistically significant.

## Results

### Overexpression of E2F family members in cervical cancer

**Fig. 1:**
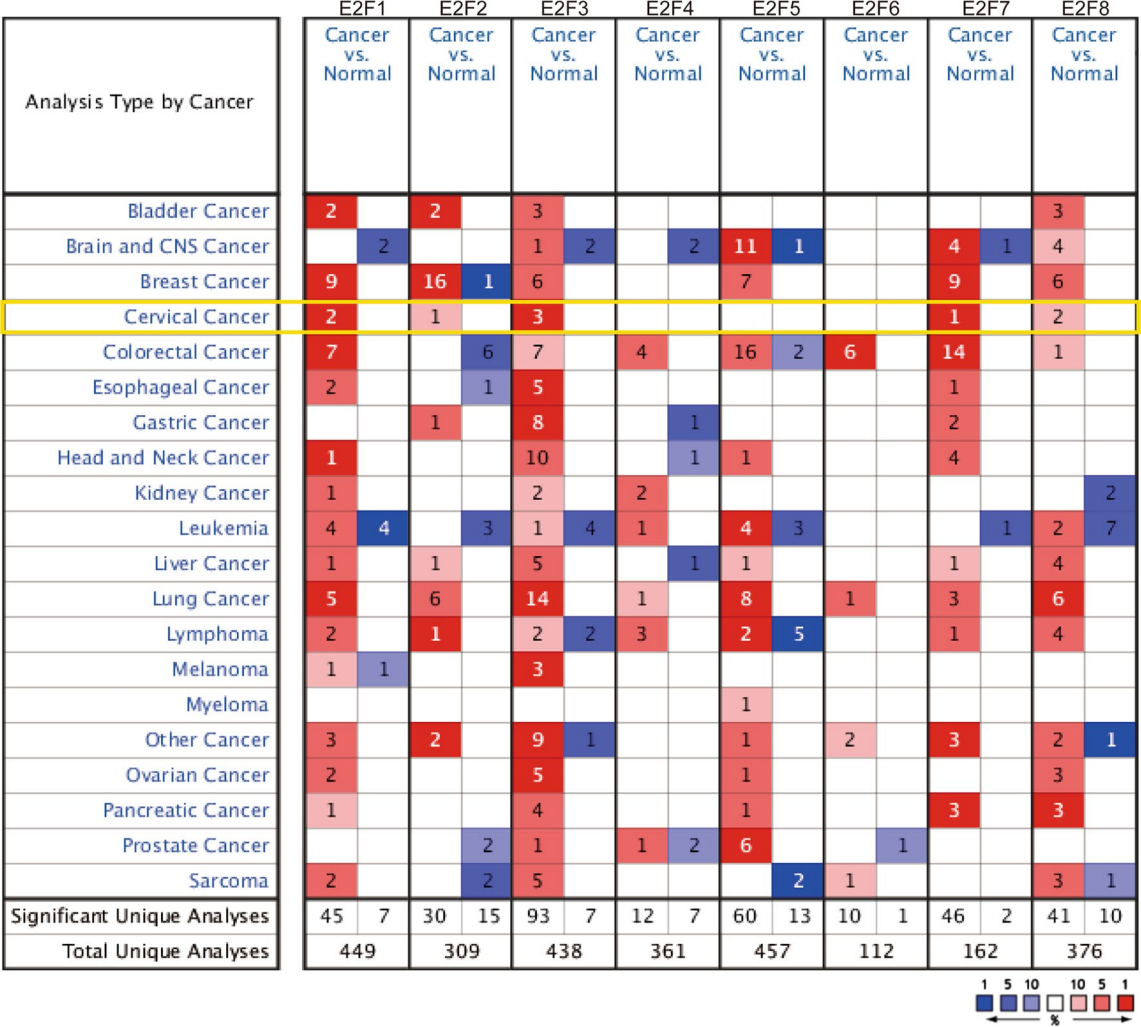
Transcriptional expression of E2Fs in different types of cancer using ONCOMINE. Differences in transcriptional expression were evaluated by Students’ t-test, using the following criteria: *p* < 0.01, fold change > 1.5, gene rank: 10%, and data type: mRNA. Cell color is determined by the best gene-rank percentile for analyses within the cell

To compare mRNA and protein levels of different E2F members in cervical cancer tissues with those found in normal tissues, we used the ONCOMINE database and GEPIA dataset in addition with IHC. As revealed by our interrogation of the ONCOMINE database, Fig. 1 presents mRNA expression of the eight E2F family members in 20 types of cancer compared with that found in normal tissues. In cervical cancer, high expression of E2F1/2/8 was observed in different datasets (Table 1). The Scotto Cervix dataset [21] revealed that mRNA expression of E2F1 in cervical cancer tissue was 3.640 times higher than that found in normal cervical tissue (*p* = 1.23E−10). Similarly, we found that E2F2 mRNA expression levels were significantly increased by 2.190 times in cervical cancer tissues in the Biewenga Cervix dataset [22] (*p* = 5.83E−6). We also found increased expression for E2F8 in cervical squamous cell carcinoma tissues compared with that found in normal cervical tissues with mRNA fold changes of 2.557 (*p* = 9.81E−5) and 2.751 (*p* = 1.96E−5) in the Zhai Cervix [23] and Scotto Cervix [21] datasets, respectively.

**Table 1:** Significant mRNA expression changes of E2Fs between cervical cancer and normal cervix tissues using ONCOMINE

	Type of cervical cancer	Fold change	*p*	t-test	Dataset (Reference)
E2F1	Cervical Squamous Cell Carcinoma	3.640	1.23E−10	7.886	Scotto Cervix [21]
E2F2	Cervical Squamous Cell Carcinoma	2.190	5.83E−6	8.117	Biewenga Cervix [22]
E2F8	Cervical Squamous Cell Carcinoma	2.557	9.81E−5	4.719	Zhai Cervix [23]
	Cervical Squamous Cell Carcinoma	2.751	1.96E−5	4.646	Scotto et al. [21]

*E2F* E2F transcription factor

**Fig. 2:**
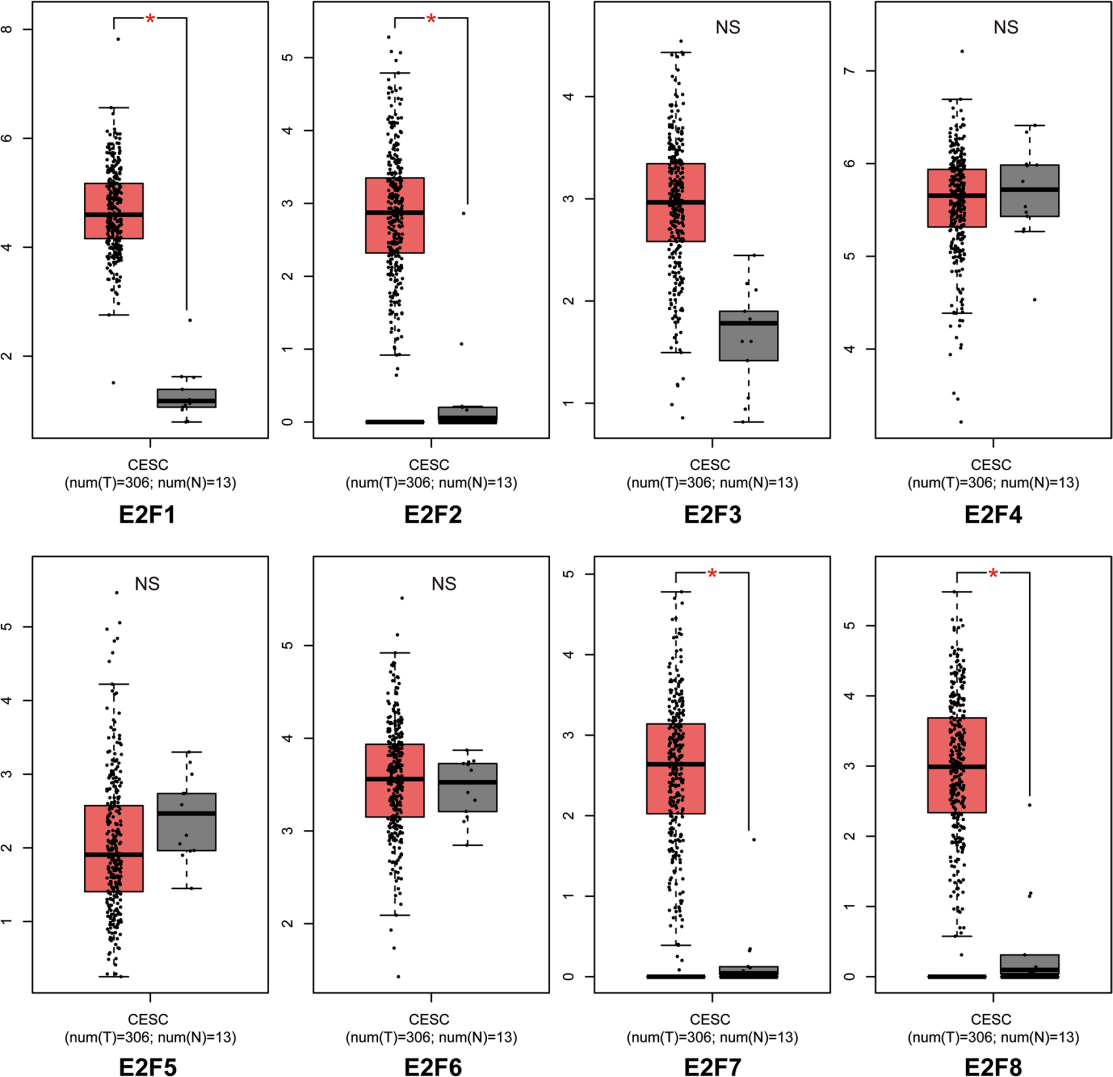
mRNA expression levels of E2Fs in cervical cancer using GEPIA. Transcriptional expression of E2F family members in cervical cancer tissues was higher than that found in normal samples as determined by Student’s t-test. *p < 0.05

Next, we confirmed the transcriptional levels of E2F family members in cervical cancer and normal tissues using the GEPIA dataset (Fig. 2) for those members that showed differences between these tissues in the ONCOMINE database. We found that mRNA expression of E2F1/2/7/8 was significantly upregulated in cervical cancer tissues compared with that found in normal tissues (all *p* < 0.05), whereas no differences in mRNA levels were found for E2F3/4/5/6.

**Fig. 3:**
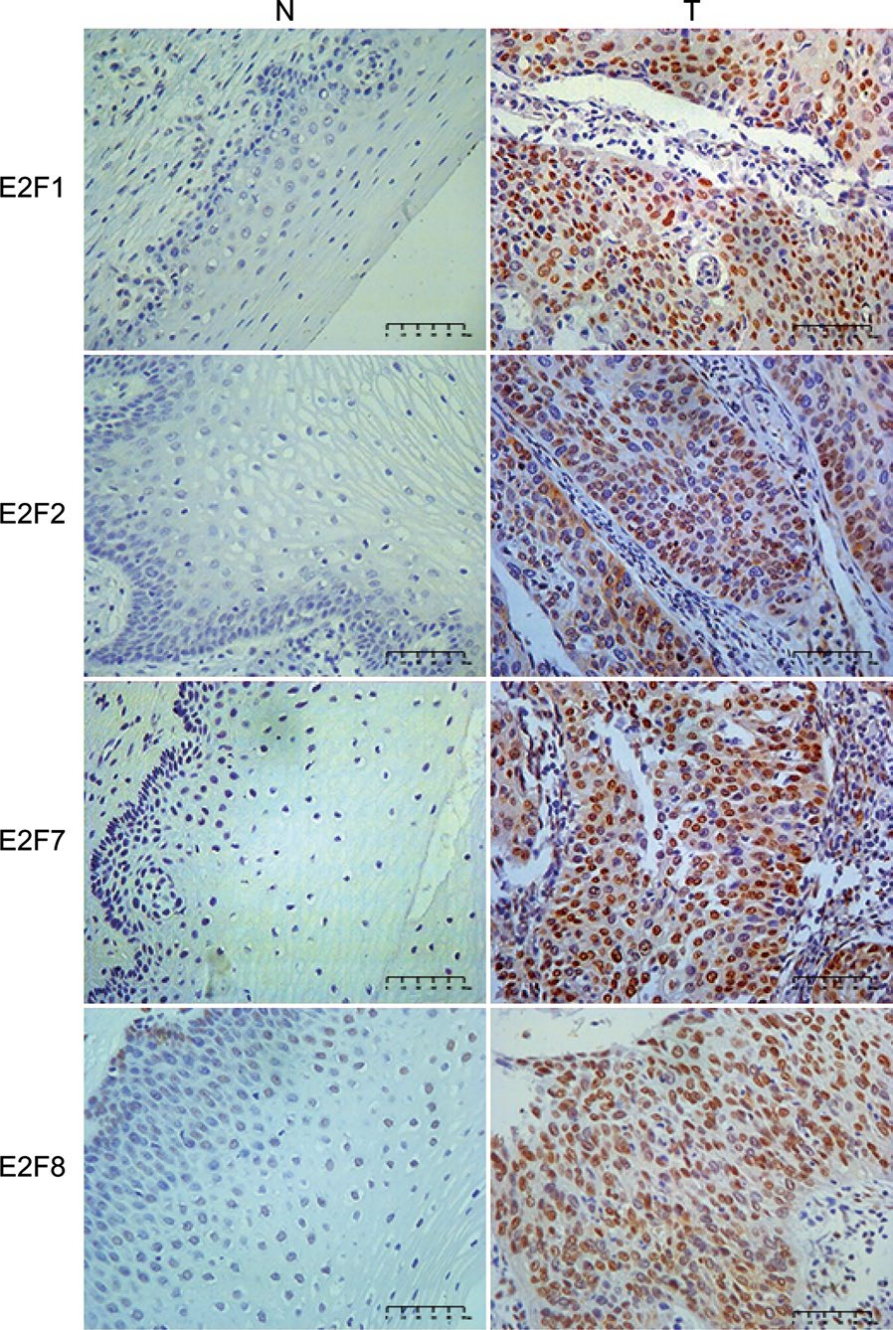
IHC of E2Fs in normal (N) and tumor (T) tissues from patients with cervical cancer. E2F1/2/7/8 expression is higher in cervical squamous cell carcinoma tissues compared with normal tissues (400 × magnification)

After exploring mRNA expression levels of E2Fs in silico, we next attempted to confirm increased E2F1/2/7/8 protein levels in tumor tissue of patients with cervical cancer by IHC. As shown in Fig. 3, E2F1/2/7/8 were highly expressed in cervical squamous cell carcinoma tissues, with no detectable expression found in normal tissues. Furthermore, E2F1/2/7/8 were mainly localized in the nuclei of tumor cells.

### Relationship between E2Fs and clinicopathological characteristics of patients with cervical cancer

To further explore the clinical significance of the expression of E2F family members in cervical squamous cell carcinoma, we determined whether there was an association between expression at the mRNA and protein levels and various clinicopathological characteristics. As shown in Additional file 1: Table S1, E2F1 expression was significantly related to histological grade (*p* = 0.01), lymph node metastasis (*p* = 0.005), lymph vessel invasion (*p* = 0.029), and depth of invasion of cervical stroma (*p* < 0.001), whereas age and tumor size were not significantly associated (both *p* > 0.05). Similar to that found for E2F1, upregulated expression of E2F2 was also significantly associated with histological grade (*p* = 0.003), lymph node metastasis (*p* = 0.004), lymph vessel invasion (*p* = 0.014), and invasion depth of cervical stroma (*p* = 0.002) (Additional file 1: Table S1). Although E2F7 expression was not associated with age, tumor size, or lymph node metastasis, there was evidence of an association with histological grade (*p* < 0.001), lymph vessel invasion (*p* = 0.003), and depth of invasion of cervical stroma (*p* < 0.001) (Additional file 1: Table S1). Regarding E2F8, there was no evidence of an association with age, tumor size, lymph node metastasis, or lymph vessel invasion; however, E2F8 expression was significantly associated with histological grade (*p* = 0.006) and invasion depth of cervical stroma (*p* = 0.004) (Additional file 1: Table S1).

### Prognostic value of E2F proteins in patients with cervical cancer

**Fig. 4:**
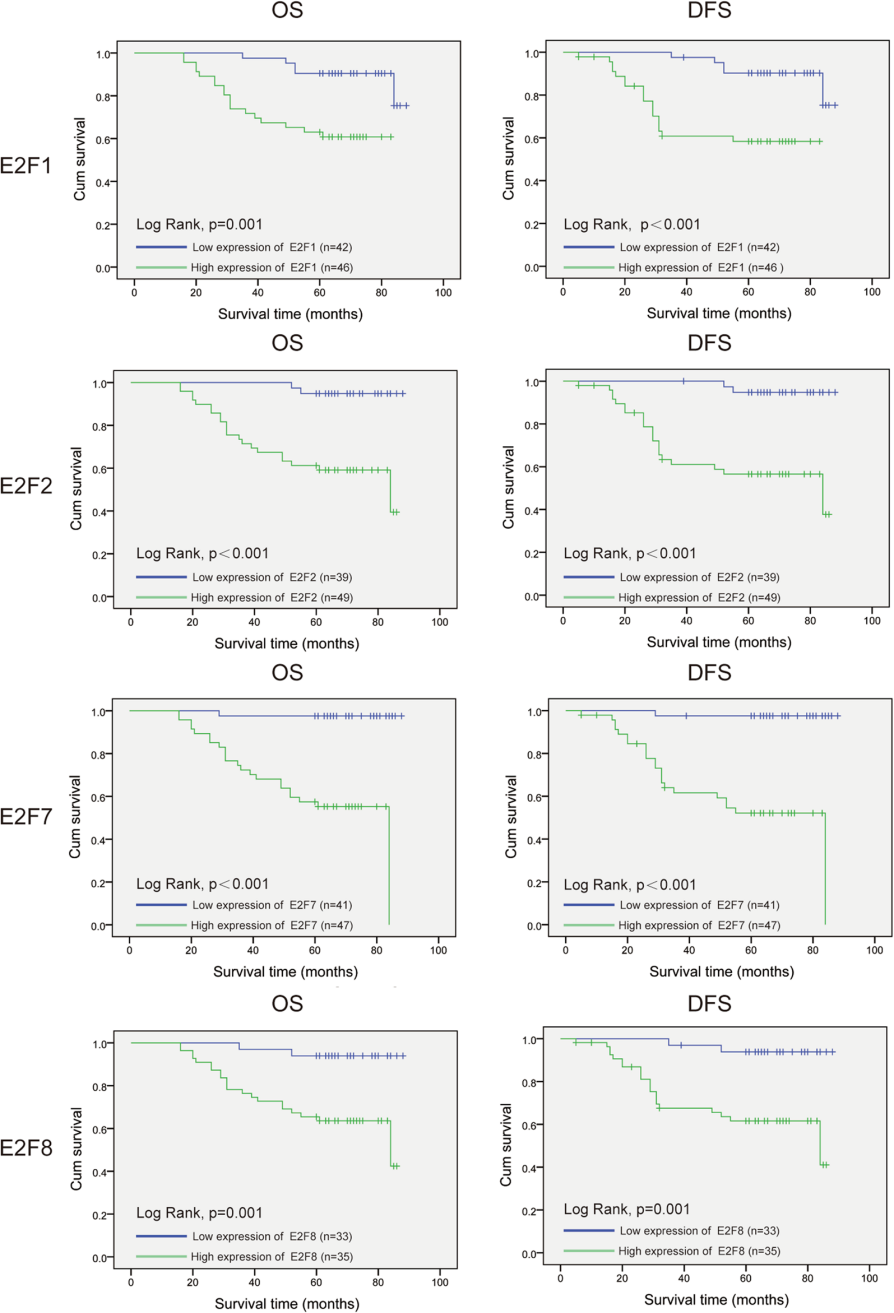
Prognostic value of E2Fs in patients with cervical cancer using Kaplan–Meier survival analysis

To evaluate the prognostic value of E2F proteins, OS and DFS were explored using Kaplan–Meier method. We found by log-rank test that increased expression of E2F1 (*p* = 0.001), E2F2 (*p* < 0.001), E2F7 (*p* < 0.001), and E2F8 protein (*p* = 0.001) was associated with significantly shorter OS compared to those in the low-expression group (Fig. 4, left panel). In terms of DFS, patients with increased E2F1 (*p* < 0.001), E2F2 (*p* < 0.001), E2F7 (*p* < 0.001), and E2F8 (*p* = 0.001) tumor expression have a poorer prognosis (Fig. 4, right panel).

Owing to an association between high E2F protein expression and poor prognosis, we further evaluated the independent prognostic value of the expression of E2Fs on OS in our cohort of patients with cervical cancer. In univariate analysis, we found that high histological grade (hazard ratio (HR) = 6.38, 95% confidence interval (CI): 2.855–14.026, *p* < 0.001), positivity for lymph vessel invasion, and high protein expression of E2F1 (HR = 5.141, 95% CI: 1.738–15.211, *p* = 0.003), E2F2 (HR = 10.668, 95% CI: 2.503–45.644, *p* = 0.001), E2F7 (HR = 27.611, 95% CI: 3.669–207.762, *p* = 0.001), and E2F8 (HR = 5.141, 95% CI: 1.738–15.211, *p* = 0.003) were related to shorter OS of patients with cervical squamous cell carcinoma (Additional file 2: Tables S2–S5, left table). Further, multivariate analysis showed that high protein expression levels of E2F1 (HR = 3.51, 95% CI: 1.177–10.469, *p* = 0.024), E2F2 (HR = 5.038, 95% CI: 1.145–22.168, *p* = 0.032), E2F7 (HR = 8.443, 95% CI: 1.089–65.443, *p* = 0.041), and E2F8 (HR = 4.393, 95% CI: 1.017–18.975, *p* = 0.047) were independently associated with significantly shorter OS of patients with cervical cancer (Additional file 2: Tables S2–S5, right table).

### Genetic mutations in E2Fs and association with OS and DFS

**Fig. 5:**
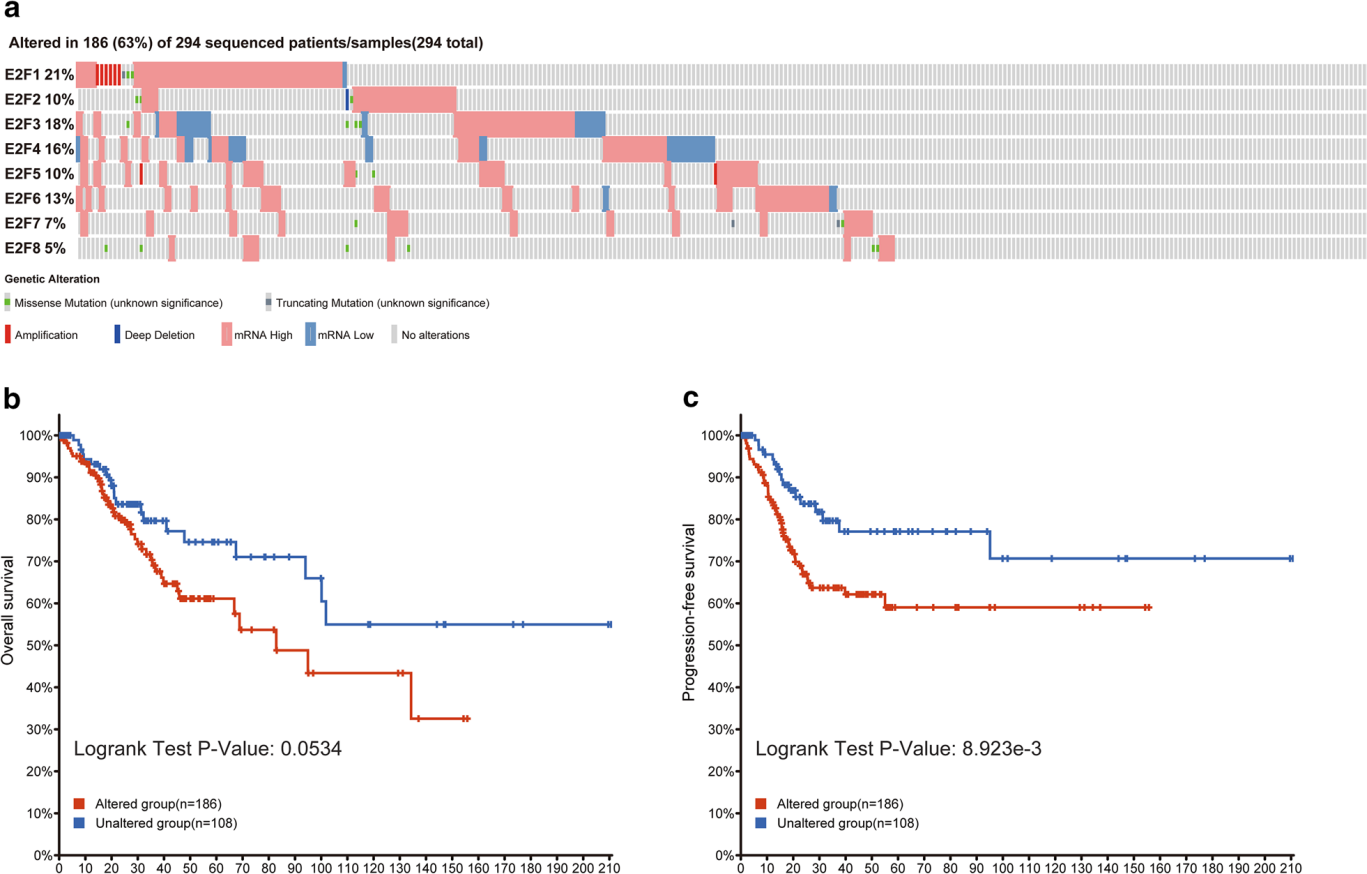
Detection of E2F gene expression and genetic mutation rate in cervical cancer. **a** In silico detection of E2F gene expression and genetic mutations in cervical cancer using cBioPortal. **b** OS analysis with Kaplan–Meier plots of patients with and without E2F genetic mutations using cBioPortal. **c** Kaplan–Meier analysis for DFS in patients with and without E2F genetic mutations using cBioPortal

Next, we explored genetic mutations in E2Fs and determined whether there was evidence of a relationship with patient OS and DFS. We found that members of the E2F family have a high mutation rate in patients with cervical cancer (Fig. 5a). Among 294 sequenced cervical cancer samples, genetic alterations occurred in 186 patients, indicating a mutation rate of 63%. *E2F1*, *E2F3*, *E2F4*, and *E2F6* showed high mutation rates of 21, 18, 16, and 13%, respectively. However, Kaplan–Meier plots showed no effect on OS (Fig. 5b), whereas the DFS of patients with cervical cancer decreased with high genetic alterations of E2Fs (log-rank test *p* = 8.923E−3) (Fig. 5c). These findings suggest that genetic factors of E2Fs also significantly affect disease progression of patients with cervical cancer.

### Functional enrichment analysis of E2Fs in patients with cervical cancer

**Fig. 6:**
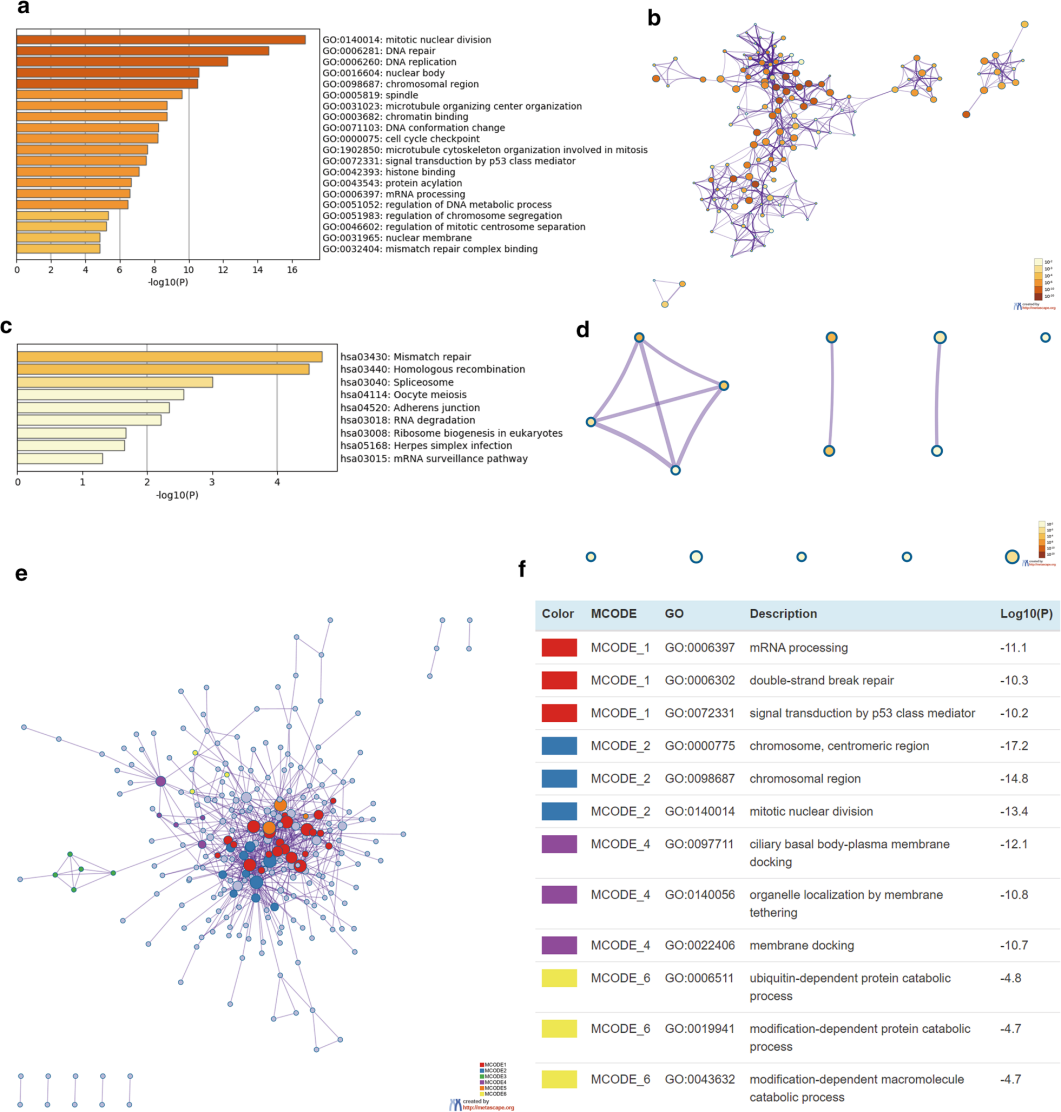
Enrichment analysis of genes with similar expression to E2Fs in cervical cancer using Metascape.** a** Heatmap of GO-enriched terms colored to reflect p-values.** b** Network of GO-enriched terms colored to reflect p-values, where terms containing more genes tend to have lower p-values.** c** Heatmap of KEGG-enriched terms colored to reflect p-values.** d** Network of KEGG enriched terms colored to reflect* p*-values; terms containing more genes tend to have lower* p*-values.** e** Protein–protein interaction network and the four most significant MCODE components from the network.** f** Independent functional enrichment analysis of the four MCODE components

We obtained 400 genes most relevant to E2F family members in cervical cancer using GEPIA. A comprehensive understanding of the biological functions of these genes may help elucidate the underlying mechanisms of action of E2Fs in cervical cancer. These 400 genes were uploaded to Metascape, and a custom analysis was performed. The top 20 GO enrichment items were classified into three functional groups: biological process (13 items), cellular component (4 items), and molecular function (3 items) (Fig. 6a, b, and Table 2). Genes relevant to E2Fs in cervical cancer were particularly associated with mitotic nuclear division, DNA repair, DNA replication, microtubule organizing center organization, mRNA processing, regulation of DNA metabolic process, regulation of chromosome segregation, regulation of mitotic centrosome separation, microtubule cytoskeleton organization involved in mitosis, signal transduction by p53 class mediator protein acylation, cell cycle checkpoint, DNA conformation change, chromosomal region, spindle, nuclear membrane, nuclear body, chromatin binding, histone binding, and mismatch repair complex binding. As shown in Fig. 6c, d, and Table 3, nine KEGG pathways were significantly associated with genes relevant to E2Fs in cervical cancer.

**Table 2:** GO function enrichment analysis of the genes relevant to E2Fs in cervical cancer using metascape

GO	Category	Description	Count	%	Log10(*P*)	Log10(*q*)
GO:0140014	GO Biological Processes	Mitotic nuclear division	30	8.24	−16.76	−12.41
GO:0006281	GO Biological Processes	DNA repair	39	10.71	−14.64	−10.77
GO:0006260	GO Biological Processes	DNA replication	25	6.87	−12.26	−8.76
GO:0031023	GO Biological Processes	Microtubule organizing center organization	15	4.12	−8.72	−5.62
GO:0006397	GO Biological Processes	mRNA processing	25	6.87	−6.58	−3.84
GO:0051052	GO Biological Processes	Regulation of DNA metabolic process	23	6.32	−6.46	−3.75
GO:0051983	GO Biological Processes	Regulation of chromosome segregation	10	2.75	−5.33	−2.76
GO:0046602	GO Biological Processes	Regulation of mitotic centrosome separation	4	1.1	−5.22	−2.68
GO:1902850	GO Biological Processes	Microtubule cytoskeleton organization involved in mitosis	14	3.85	−7.59	−4.67
GO:0072331	GO Biological Processes	Signal transduction by p53 class mediator	19	5.22	−7.53	−4.63
GO:0043543	GO Biological Processes	Protein acylation	17	4.67	−6.64	−3.87
GO:0000075	GO Biological Processes	Cell cycle checkpoint	18	4.95	−8.19	−5.15
GO:0071103	GO Biological Processes	DNA conformation change	22	6.04	−8.24	−5.17
GO:0098687	GO Cellular Components	Chromosomal region	26	7.14	−10.5	−7.23
GO:0005819	GO Cellular Components	Spindle	25	6.87	−9.6	−6.36
GO:0031965	GO Cellular Components	Nuclear membrane	16	4.4	−4.84	−2.37
GO:0016604	GO Cellular Components	Nuclear body	40	10.99	−10.57	−7.24
GO:0003682	GO Molecular Functions	Chromatin binding	30	8.24	−8.72	−5.62
GO:0042393	GO Molecular Functions	Histone binding	16	4.4	−7.1	−4.28
GO:0032404	GO Molecular Functions	Mismatch repair complex binding	4	1.1	−4.81	−2.35

**Table 3:** KEGG function enrichment analysis of the genes relevant to E2Fs in cervical cancer using metascape

GO	Category	Description	Count	%	Log10*(P*)	Log10(*q*)
hsa03430	KEGG Pathway	Mismatch repair	5	1.37	−4.69	−2.1
hsa03440	KEGG Pathway	Homologous recombination	6	1.65	−4.49	−2.1
hsa03040	KEGG Pathway	Spliceosome	8	2.2	−3	−1.01
hsa04114	KEGG Pathway	Oocyte meiosis	7	1.92	−2.56	−0.71
hsa04520	KEGG Pathway	Adherens junction	5	1.37	−2.33	−0.54
hsa03018	KEGG Pathway	RNA degradation	5	1.37	−2.21	−0.47
hsa03008	KEGG Pathway	Ribosome biogenesis in eukaryotes	5	1.37	−1.67	−0.03
hsa05168	KEGG Pathway	Herpes simplex infection	7	1.92	−1.65	−0.03
hsa03015	KEGG Pathway	mRNA surveillance pathway	4	1.1	−1.31	0

To obtain a better understanding of the correlations between these genes relevant to E2Fs in cervical cancer, we analyzed protein–protein interactions through Metascape. The results of the protein–protein interaction network and MCODE components are shown in Fig. 6e, f. Within the protein–protein interaction network, the six most significant MCODE components were extracted, and pathway and process enrichment analyses were independently performed on each MCODE component. We found a particular association with the biological functions of mRNA processing, double-strand break repair, signal transduction by p53 class mediator, centromeric region, chromosomal region, mitotic nuclear division, ciliary basal body-plasma membrane docking, organelle localization by membrane tethering, membrane docking, ubiquitin-dependent protein catabolic process, modification-dependent protein catabolic process, and modification-dependent macromolecule catabolic process.

### Expression of E2F2 and E2F7 in cervical cancer tissues and localization in HeLa and C-33 A cells

**Fig. 7:**
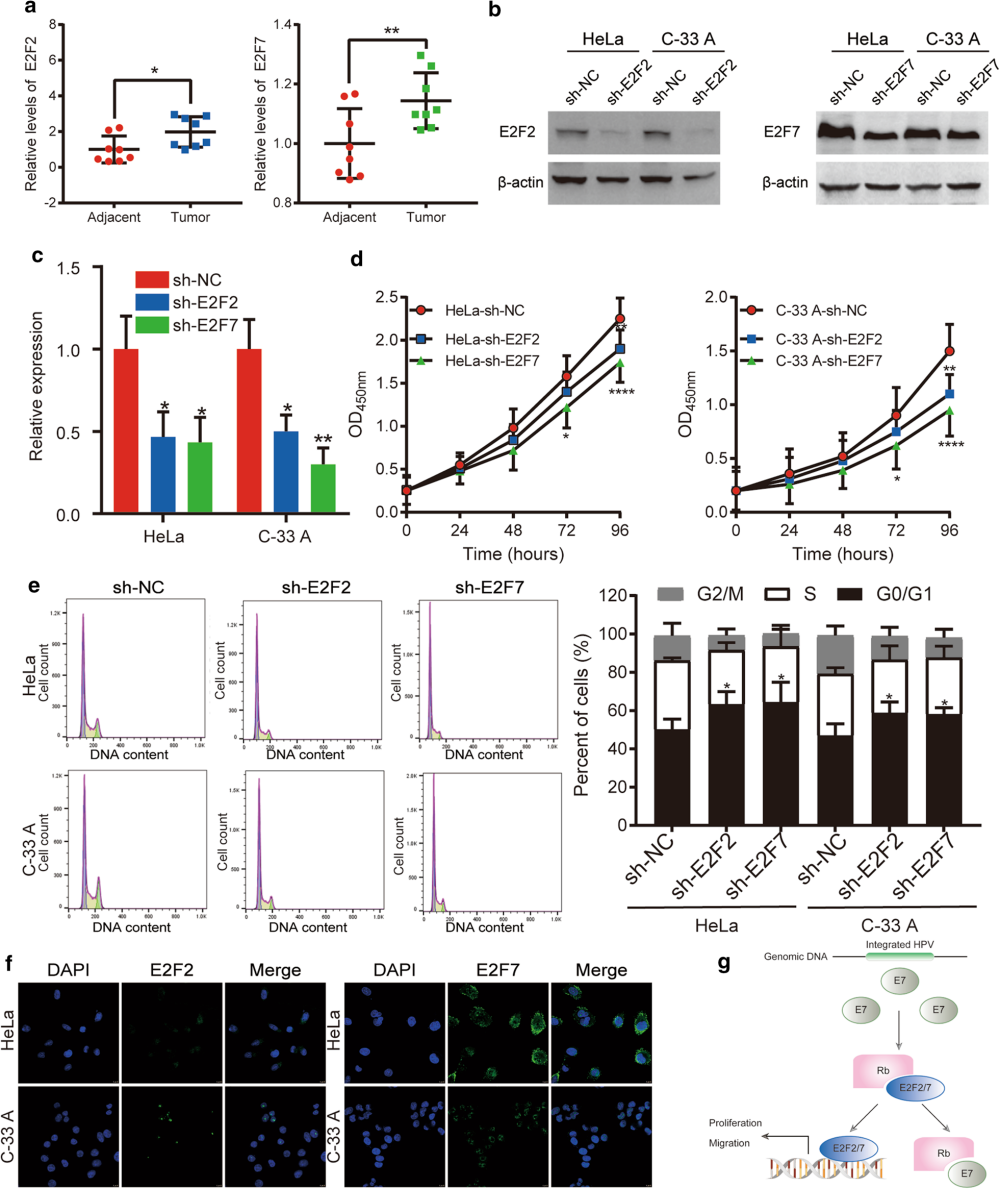
Knockdown of E2F2 and E2F7 suppresses cell proliferation and induces cell cycle arrest.** a** E2F2 and E2F7 expression was increased in cervical cancer tissues compared with adjacent normal tissues.** b**,** c** Evaluation of E2F2 and E2F7 knockdown efficiency in HeLa and C-33 A cells by Western blot and RT-PCR.** d** Immunofluorescent localization of E2F2 and E2F7 in HeLa and C-33 A cells.** e** Cell proliferation was determined in HeLa and C-33 A cells by CCK-8 assay.** f** Cell cycle analysis of HeLa and C-33 A cells by flow cytometry. Data are expressed as the mean ± SD (n = 3). **g** Proposed model for E2F2- and E2F7-induced proliferation and migration in cervical cancer. The human papillomavirus E7 oncogene, which abrogates RB protein function, released “freed E2F2/7”, and activates E2F-regulated genes of proliferation and migration. All experiments were performed in triplicate. *p-value t-test < 0.05; **p-value t-test < 0.01; ****p-value t-test < 0.0001 versus NC group

Expression levels of E2F2 and E2F7 in cervical cancer tissues were determined by RT-qPCR. We found that expression of E2F2 (*p* < 0.05) and E2F7 (*p* < 0.01) was significantly upregulated in primary cervical cancer tissues compared with adjacent normal tissues (Fig. 7a). Further, based on immunofluorescence assay, we found that E2F2 and E2F7 were located in the nuclei of C-33 A cells, whereas E2F2 and E2F7 located both in the nucleus and in cytoplasm of HeLa cells (Fig. 7f). We then investigated the Human Protein Atlas database and found that E2F7 was located in the nucleus of HeLa cells, which was not consistent with our observation and needed to be further explored and confirmed.

### Efficiency of E2F2 and E2F7 knockdown

HeLa and C-33 A cells were transfected with sh-E2F2 and sh-E2F7. RT-qPCR and western blot analysis were performed to assess the effect of E2F2 and E2F7 knockdown. Our findings show that expression levels of E2F2 and E2F7 in cells transfected with sh-E2F2 and sh-E2F7, were significantly decreased compared with NC HeLa and C-33 A cells (Fig. 7b, c).

### Effects of E2F2 and E2F7 knockdown on proliferation, migration, and stemness of HeLa and C-33 A cells

Because expression of E2Fs was associated with lymph node metastasis, lymph vessel invasion, and depth of invasion of cervical stroma, we further evaluated the effect of E2Fs on tumorigenic activity of cervical cancer cells. First, we observed cell cycle changes through knockdown of E2F2 and E2F7 in HeLa and C-33 A cells, and determined the percentage of cells in G0/G1, S, and G2/M phase by flow cytometry. Compared to control groups, we found that cells in the G0/G1 phase were significantly increased in sh-E2F2- and sh-E2F7-transfected HeLa and C-33 A cells (*p* < 0.05). Thus, E2F2 and E2F7 promote cervical cancer cell proliferation through regulating the cell cycle (Fig. 7e). Next, the inhibitory effect of E2F2 and E2F7 knockdown on cervical cancer cell growth was validated by CCK-8 analysis. After E2F2 and E2F7 knockdown, we found that the number of cells in sh-E2F2- and sh-E2F7-transfected groups was significantly lower compared to that found in sh-NC-transfected HeLa and C-33 A cells (Fig. 7d). These results confirm that E2F2 and E2F7 markedly suppress proliferation of HeLa and C-33 A cells.

**Fig. 8:**
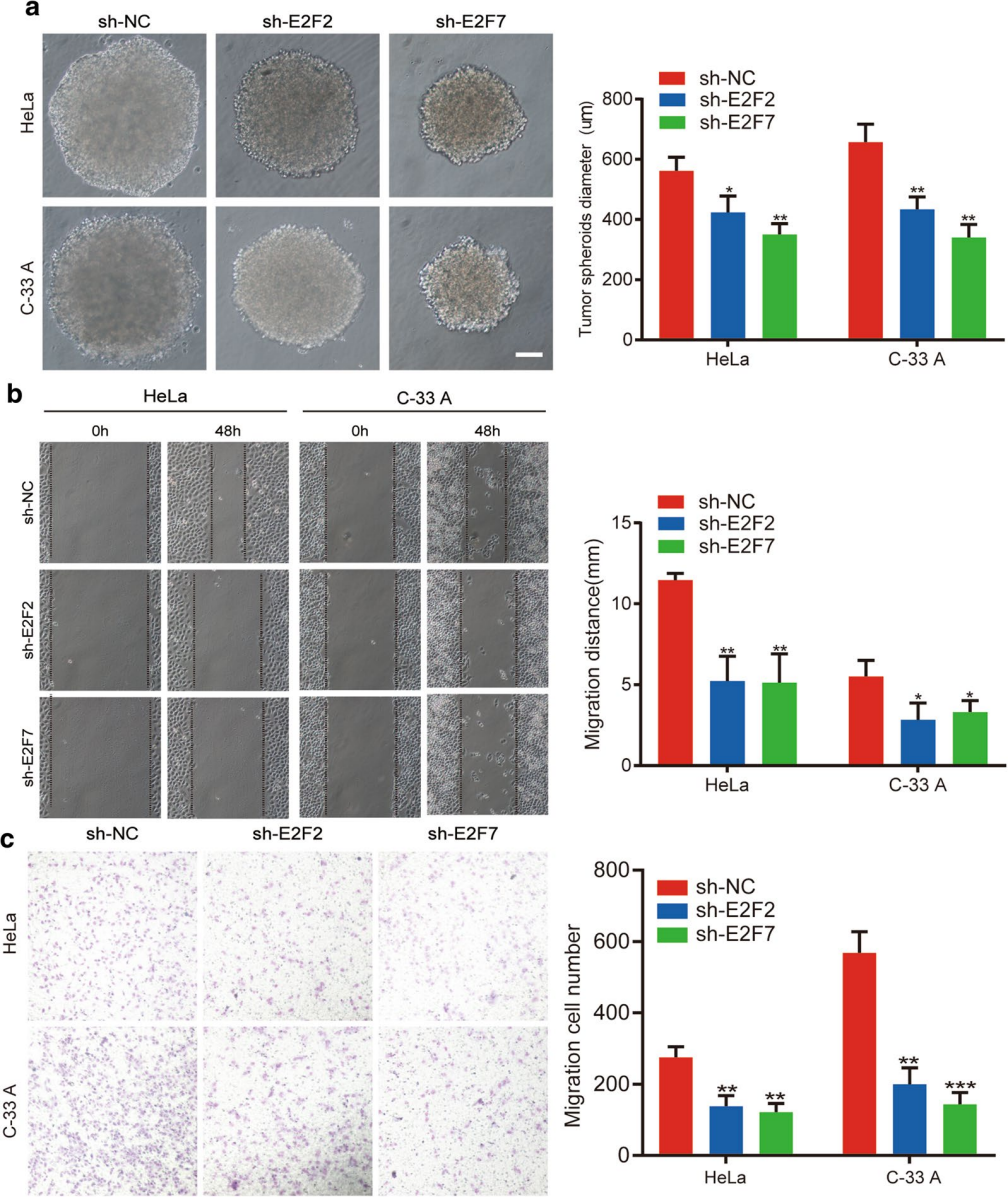
Knockdown of E2F2 and E2F7 inhibits cell migration and stemness.** a**,** b** Cell migration was determined in HeLa and C-33 A cells by wound healing and Transwell assays.** c** Graph reporting the diameter of spheres formed by sphere-forming assay (scale bar; 50 μm). All experiments were performed in triplicate. *p-value t-test < 0.05; **p-value t-test < 0.01; ***p-value t-test < 0.001 versus NC group

We then evaluated the migration capacity of HeLa- and C-33 A-transfected cells via in vitro wound healing and Transwell migration assays. Our Transwell assay results show that cell migration was significantly reduced in cells transfected with sh-E2F2 or sh-E2F7 compared with NC cells (Fig. 8c). Furthermore, based on comparisons of migration distance at 0 versus 48 h, we found using wound healing assays that migration ability was diminished following E2F2 and E2F7 knockdown (Fig. 8b). We conclude that downregulation of E2F2 and E2F7 results in a decrease in visible metastases.

HeLa and C-33 A cells were cultured in serum-free medium for 5 days for spheroid formation, and we found that the diameter of tumor microspheres in sh-E2F2- and sh-E2F7-transfected HeLa and C-33 A cells was shorter than that found in the control group (Fig. 8a). These results indicate that reduced E2F2 and E2F7 expression inhibit properties of tumor microsphere stemness.

## Discussion

To our knowledge, this study is the first to report increased expression of E2F1/2/7/8 in cervical cancer and provide evidence of an association with higher histological grade, lymph node metastasis, lymph vessel invasion, and deep invasion of cervical stroma. We also found that upregulation of E2F1/2/7/8 is an independent prognostic factor for patient OS and that genetic alterations of E2Fs were associated with poor DFS in cervical cancer, indicating that dysregulation of E2F1/2/7/8 may be associated with progression of cervical cancer. Because an oncogenic role of E2F1 and E2F8 in cervical cancer has been reported [24, 25], we knocked down E2F2 and E2F7 by shRNA in cells from the cervical cancer cell lines HeLa and C-33 A, and further investigated to assess the effect of E2F2 and E2F7 knockdown on cellular functions of HeLa and C-33 A cells. We discovered that E2F2 and E2F7 knockdown suppressed proliferation and migration abilities, promoted cell cycle arrest, and inhibited stemness of both HeLa and C-33 A cells in vitro, suggesting that E2F2 and E2F7 may function as oncogenes, leading to tumor progression or metastasis of HPV-positive and HPV-negative cervical cancer.

In line with the possible role of E2Fs in human tumorigenesis, previous studies have shown overexpression of E2Fs in several human malignant tumors at both the mRNA and protein level [26–31]. Importantly, in the current study, we show evidence of an association between E2F1/2/7/8 with histological grade, lymph node metastasis, lymph vessel invasion, and deep invasion of cervical stroma. Other studies have similarly reported an association between increased expression of E2Fs and high-risk clinicopathological factors in lung carcinoma [32], breast cancer [7], clear cell renal cell carcinoma [33], and non-muscle invasive bladder cancer [34]. In ovarian cancer, it was found that E2F1/2/5/8 are poor prognostic biomarkers and therapeutic targets [35]]; in contrast, Yao et al. demonstrated that E2F1/2/3/5/7/8 are potential biomarkers for the diagnosis of colon cancer and E2F3/4/7/8 are potential targets of precision therapy [28]. In the current study, we found that genetic mutations in E2Fs are associated with poor DFS in cervical cancer, a finding that may be accounted for by Shan et al. [36]. They demonstrated that point mutations in *E2F1* resulted in a failure of protein binding to retinoblastoma protein (Rb), which functions as a negative regulator of cell proliferation. Without interacting with Rb, mutated E2F1 increases cell cycle progression [36]. Dimova et al. also demonstrated that alterations such as point mutations, deletions, amplifications or promoter methylation in components of the RB pathway including *E2Fs* often occur in human tumors. These alterations can be either inactivating or activating [37]. Collectively, these findings indicate that E2Fs played a crucial role in human tumorigenesis and tumor progression.

Because expression of E2Fs is associated with multiple clinicopathological factors, including lymph node metastasis, lymph vessel invasion, and depth of invasion of cervical stroma, we further investigated the role of E2F2 and E2F7 in the processes of cell proliferation, migration, and stemness in cervical cancer cells. We found that E2F2 and E2F7 knockdown inhibited cell proliferation, migration, and stemness. Considering that E2F2 and E2F7 downregulation inhibits proliferation of cervical cancer cells, we performed cell cycle analysis to evaluate the role of E2F2 and E2F7 in cell-cycle distribution. Our findings showed that E2F2 and E2F7 knockdown induced cell cycle arrest in the G0/G1 phase in cervical cancer cells. Consistent with our findings, Nakahata et al. demonstrated a tumorigenic role of E2F2 in vitro and in vivo, suggesting E2F2 is closely involved in gliomagenesis and may be a potential therapeutic target in malignant gliomas [38]. Further, an in vivo study showed that downregulation of E2F2 inhibits features of stemness and decreases the cancer stem cell population in lung cancer [39]. In addition, inhibition of E2F2 by miR-638 downregulates the proportion of CD24−/CD44 + cells and levels of SOX2 and OCT4 [30]. Regarding E2F7, Wang et al. verified that inhibition of E2F7 expression in cells from prostate cancer cell lines dramatically decreased cell proliferation, increased cell cycle arrest in the G1 phase, and resulted in higher apoptotic rates compared with those in NC groups [29]. It was also found that downregulation of E2F7 by miRNA-302a/d decreased proliferation of hepatocellular carcinoma cells and significantly inhibited stemness of lung cancer stem cells by targeting the E2F7/AKT/β-catenin/CCND1 signaling pathway [31]. Clearly, further studies on the mechanisms underlying the roles of E2F2 and E2F7 in cell proliferation and migration are warranted.

## Conclusions

In this study, we comprehensively explored the transcriptional expression of E2Fs in cervical cancer, finding that E2F1/2/7/8 are significantly overexpressed in this disease. We then confirmed these findings using IHC and RT-qPCR at the protein and mRNA level, and using in vitro experiments, showed that E2F2 and E2F7 are involved in cell proliferation, migration, and cell cycle regulation in both HPV-positive and HPV-negative cervical cancer cells. We also assessed the prognostic import of E2F1/2/7/8 in patients with cervical cancer by analyzing clinicopathological data. Furthermore, we showed that high expression of E2F1/2/7/8 proteins was significantly associated with shorter OS and DFS in patients with cervical cancer. Using multivariate analysis, we also confirmed E2F1/2/7/8 as independent prognostic factors for shorter OS of patients with cervical cancer. These results indicate that E2F1, E2F2, E2F7, and E2F8 may serve as prognostic biomarkers and potential therapeutic targets for cervical cancer.

## Supplementary information

**Additional file 1: Table S1.** Association between E2F1/2/7/8 protein expression and clinicopathological parameters in cervical cancer.

**Additional file 2: Table S2.** Univariate and multivariate analyses of overall survival in patients with cervical squamous cell carcinoma.** Table S3.** Univariate and multivariate analyses of overall survival in patients with cervical squamous cell carcinoma.** Table S4.** Univariate and multivariate analyses of overall survival in patients with cervical squamous cell carcinoma.** Table S5.** Univariate and multivariate analyses of overall survival in patients with cervical squamous cell carcinoma.

## Data Availability

Not applicable.

## Abbreviations

E2Fs: E2F transcription factors
GEPIA: Gene Expression Profiling Interactive Analysis
OS: Overall survival
DFS: Disease-free survival
HPV: Human papilloma virus
GTEx: Genotype Tissue Expression
KEGG: Kyoto Encyclopedia of Genes and Genomes
GO: Gene ontology
GSEA: Gene Set Enrichment Analysis
IHC: Immunohistochemistry
MCODE: Molecular complex detection
Rb: Retinoblastoma protein
GISTIC: Genomic Identification of Significant Targets in Cancer
shRNA: Short hairpin RNA
RT-qPCR: Quantitative reverse transcription polymerase chain reaction
PBS: Phosphate-buffered saline
FBS: Fetal bovine serum
FIGO: International Federation of Gynecology and Obstetrics
