# Supplementary material for: E2F1/2/7/8 as independent indicators of survival in patients with cervical squamous cell carcinoma
Source: Cancer Cell Int. 2020 Oct 12;20:500. doi: 10.1186/s12935-020-01594-0 (PMC7552358; doi:10.1186/s12935-020-01594-0)
Supplement: Supplementary file 1 — Additional file 1: Table S1. Association between E2F1/2/7/8 protein expression and clinicopathological parameters in cervical cancer. [file 12935_2020_1594_MOESM1_ESM.doc]

Table S1. Association between E2F1/2/7/8 protein expression and clinicopathological parameters in cervical cancer.

| **Variables** | **E2F1** | |  | **E2F2** | |  | **E2F7** | |  | **E2F8** | |  |
| --- | --- | --- | --- | --- | --- | --- | --- | --- | --- | --- | --- | --- |
|  | **Low expression** | **High expression** | ***p* value** | **Low expression** | **High expression** | ***p* value** | **Low expression** | **High expression** | ***p* value** | **Low expression** | **High expression** | ***p* value** |
| **Age (y)** |  |  |  |  |  |  |  |  |  |  |  |  |
| <43 | 16 | 26 | 0.084 | 16 | 26 | 0.261 | 20 | 22 | 0.853 | 15 | 27 | 0.741 |
| ≥43 | 26 | 20 |  | 23 | 23 |  | 21 | 25 |  | 18 | 28 |  |
| **Clinical stage** |  |  |  |  |  |  |  |  |  |  |  |  |
| I | 31 | 19 | 0.002 | 25 | 25 | 0.218 | 26 | 24 | 0.243 | 26 | 24 | 0.001 |
| II | 11 | 27 |  | 14 | 24 |  | 15 | 23 |  | 7 | 31 |  |
| **Histological grade** |  |  |  |  |  |  |  |  |  |  |  |  |
| G1 | 22 | 10 | 0.010 | 19 | 13 | 0.003 | 22 | 10 | 0.000 | 17 | 15 | 0.006 |
| G2 | 11 | 17 |  | 15 | 13 |  | 15 | 13 |  | 12 | 16 |  |
| G3 | 9 | 19 |  | 5 | 23 |  | 4 | 24 |  | 4 | 24 |  |
| **Tumor size** |  |  |  |  |  |  |  |  |  |  |  |  |
| <4cm | 26 | 24 | 0.357 | 20 | 30 | 0.350 | 26 | 24 | 0.243 | 26 | 24 | 0.243 |
| ≥4cm | 16 | 22 |  | 19 | 19 |  | 15 | 23 |  | 15 | 23 |  |
| **Lymph node metastasis** |  |  |  |  |  |  |  |  |  |  |  |  |
| No | 36 | 27 | 0.005 | 34 | 29 | 0.004 | 33 | 30 | 0.084 | 33 | 30 | 0.084 |
| Yes | 6 | 19 |  | 5 | 20 |  | 8 | 17 |  | 8 | 17 |  |
| **Lymph vessel invasion** |  |  |  |  |  |  |  |  |  |  |  |  |
| No | 28 | 20 | 0.029 | 27 | 21 | 0.014 | 31 | 21 | 0.003 | 22 | 26 | 0.077 |
| Yes | 14 | 26 |  | 12 | 28 |  | 10 | 26 |  | 11 | 29 |  |
| **Invasion depth of cervical stroma** |  |  |  |  |  |  |  |  |  |  |  |  |
| <1/2 | 33 | 19 | 0.000 | 30 | 22 | 0.002 | 30 | 22 | 0.000 | 26 | 26 | 0.004 |
| ≥1/2 | 9 | 27 |  | 9 | 27 |  | 7 | 29 |  | 7 | 29 |  |
