# Supplementary material for: E2F1/2/7/8 as independent indicators of survival in patients with cervical squamous cell carcinoma
Source: Cancer Cell Int. 2020 Oct 12;20:500. doi: 10.1186/s12935-020-01594-0 (PMC7552358; doi:10.1186/s12935-020-01594-0)
Supplement: Supplementary file 2 — Additional file 2: Table S2. Univariate and multivariate analyses of overall survival in patients with cervical squamous cell carcinoma. Table S3. Univariate and multivariate analyses of overall survival in patients with cervical squamous cell carcinoma. Table S4. Univariate and multivariate analyses of overall survival in patients with cervical squamous cell carcinoma. Table S5. Univariate and multivariate analyses of overall survival in patients with cervical squamous cell carcinoma. [file 12935_2020_1594_MOESM2_ESM.docx]

Table S2. Univariate and multivariate analyses of overall survival in patients with cervical squamous cell carcinoma.

| **Variables** | **Univariate analysis** | | | **Multivariate analysis** | | |
| --- | --- | --- | --- | --- | --- | --- |
|  | ***p* value** | **Hazard Ratio** | **95% confidence interval** | ***p* value** | **Hazard Ratio** | **95% confidence interval** |
| **Histological grade** | 0.000 | 6.328 | 2.855-14.026 | 0.001 | 3.734 | 1.756-7.939 |
| **Lymph vessel invasion** | 0.000 | 15.566 | 3.632-66.721 | 0.009 | 7.486 | 1.651-33.949 |
| **E2F1 expression** | 0.003 | 5.141 | 1.738-15.211 | 0.024 | 3.510 | 1.177-10.469 |

Table S3. Univariate and multivariate analyses of overall survival in patients with cervical squamous cell carcinoma.

| **Variables** | **Univariate analysis** | | | **Multivariate analysis** | | |
| --- | --- | --- | --- | --- | --- | --- |
|  | ***p* value** | **Hazard Ratio** | **95% confidence interval** | ***p* value** | **Hazard Ratio** | **95% confidence interval** |
| **Histological grade** | 0.000 | 6.328 | 2.855-14.026 | 0.003 | 3.111 | 1.484-6.524 |
| **Lymph vessel invasion** | 0.000 | 15.566 | 3.632-66.721 | 0.011 | 7.072 | 1.562-32.025 |
| **E2F2 expression** | 0.001 | 10.688 | 2.503-45.644 | 0.032 | 5.038 | 1.145-22.168 |

Table S4. Univariate and multivariate analyses of overall survival in patients with cervical squamous cell carcinoma.

| **Variables** | **Univariate analysis** | | | **Multivariate analysis** | | |
| --- | --- | --- | --- | --- | --- | --- |
|  | ***p* value** | **Hazard Ratio** | **95% confidence interval** | ***p* value** | **Hazard Ratio** | **95% confidence interval** |
| **Histological grade** | 0.000 | 6.328 | 2.855-14.026 | 0.004 | 2.919 | 1.398-6.093 |
| **Lymph vessel invasion** | 0.000 | 15.566 | 3.632-66.721 | 0.013 | 6.548 | 1.484-28.896 |
| **E2F7 expression** | 0.001 | 27.611 | 3.669-207.762 | 0.041 | 8.443 | 1.089-65.443 |

Table S5. Univariate and multivariate analyses of overall survival in patients with cervical squamous cell carcinoma.

| **Variables** | **Univariate analysis** | | | **Multivariate analysis** | | |
| --- | --- | --- | --- | --- | --- | --- |
|  | ***p* value** | **Hazard Ratio** | **95% confidence interval** | ***p* value** | **Hazard Ratio** | **95% confidence interval** |
| **Histological grade** | 0.000 | 6.328 | 2.855-14.026 | 0.001 | 3.316 | 1.595-6.898 |
| **Lymph vessel invasion** | 0.000 | 15.566 | 3.632-66.721 | 0.004 | 8.687 | 1.963-38.448 |
| **E2F8 expression** | 0.003 | 5.141 | 1.738-15.211 | 0.047 | 4.393 | 1.017-18.975 |
